# Recombinant expression of SARS-CoV-2 receptor binding domain (RBD) in *Escherichia coli *and its immunogenicity in mice

**DOI:** 10.22038/IJBMS.2022.65045.14333

**Published:** 2022-09

**Authors:** Zahra Rahbar, Shahram Nazarian, Ruhollah Dorostkar, Fattah Sotoodehnejadnematalahi, Jafar Amani

**Affiliations:** 1 Department of Biology, Science and Research Branch, Islamic Azad University, Tehran, Iran; 2 Department of Biology, Imam Hossein University, Tehran, Iran; 3 Applied Virology Research Center, Baqiyatallah University of Medical Sciences, Iran; 4 Applied Microbiology Research Center, System Biology and Poisonings Institute, Baqiyatallah University of Medical Sciences, Tehran, Iran

**Keywords:** Neutralizing antibodies, RBD, Recombinant proteins, SARS-CoV-2, Vaccines

## Abstract

**Objective(s):**

The severe acute respiratory syndrome coronavirus-2 (SARS-CoV-2), giving rise to the coronavirus disease 2019 (COVID-19), has become a danger to wellbeing worldwide. Thus, finding efficient and safe vaccines for COVID-19 is of great importance. As a basic step amid contamination, SARS-CoV-2 employs the receptor-binding domain (RBD) of the spike protein to lock in with the receptor angiotensin-converting enzyme 2 (ACE2) on host cells. SARS-CoV-2 receptor-binding domain (RBD) is the main human antibody target for developing vaccines and virus inhibitors, as well as neutralizing antibodies. A bacterial procedure was developed for the expression and purification of the SARS-CoV-2 spike protein receptor-binding domain.

**Materials and Methods:**

In this research study, RBD was expressed by *Escherichia coli *and purified with Ni-NTA chromatography. Then it was affirmed by the western blot test. The immunogenicity and protective efficacy of RBD recombinant protein were assessed on BALB/c mice. Additionally, RBD recombinant protein was tested by ELISA utilizing sera of COVID-19 healing patients contaminated with SARS-CoV-2 wild type and Delta variation.

**Results:**

Indirect ELISA was able to detect the protein RBD in serum of the immunized mouse expressed in E. coli. The inactive SARS-CoV2 was detected by antibodies within the serum of immunized mice. Serum antibodies from individuals recovered from Covid19 reacted to the expressed protein.

**Conclusion:**

Our findings showed that RBD is of great importance in vaccine design and it can be used to develop recombinant vaccines through induction of antibodies against RBD.

## Introduction

The emergence of COVID-19 was caused by severe acute respiratory syndrome coronavirus 2 (SARS-CoV-2) in Wuhan, China, and quickly spread throughout the globe (1). Human coronaviruses (hCoVs) are defined as enveloped viruses with a positive-sense, single-stranded RNA genome. HCoVs genome is a large RNA virus, which ranges from 26.4 to 31.7 kilobases (2). They are divided into four genera, beta-CoV, alpha-CoV, delta-CoV, and gamma-CoV. SARS-CoV-2 is identified as a beta coronavirus genus member which includes MERS-CoV, SARS-CoV, bat SARS-related coronaviruses (SARSr-CoV), and others recognized in humans and animals. The genome of the virus encodes 16 non-structural proteins (NSP) and four main structural proteins. The structural proteins are the nucleocapsid (N) protein, the envelope (E) protein, the spike (S) protein, and the membrane (M) protein. The SARS-CoV-2 S protein possesses 1,273 amino acids, longer than that of known bat SARSr-CoVs (1,245–1,269 amino acids) and SARS-CoV (1,255 amino acids). The S protein contains two subunits (S1 & S2). Subunit S1 carries the receptor-binding domain (RBD), interacting directly with the angiotensin-converting enzyme 2 (ACE2) receptor. While the S2 subunit is responsible for the fusion of the virus envelope with the host cell membrane (2-4). The most variable region in the coronavirus spike protein is the RBD sequence which is subject to mutations. Six amino acid residues are necessary for binding to the human ACE2 receptor, this amino acid sequence in SARS-CoV included Y442, L472, N479, D480, T487, and Y4911 while in SARS-CoV2 they respectively correspond to L455, F486, Q493, S494, N501, and Y505 residues. Research has shown that five out of these six SARS-CoV-2 residues are not similar to their SARS-CoV patterns, and this change may be due to deletions, mutations, or insertions in the S1-S2 segment of coronaviruses (5). According to WHO, the new SARS-CoV-2 strains include two groups: SARS-CoV-2 variants of concern (VoCs) and SARS-CoV-2 variants of interest (VoIs). Five SARS-CoV-2 variants of concern, Alpha, Beta, Gamma, Delta, and Omicron, and two SARS-CoV-2 variants of interest, Lambda, and Mu. These variants cause changes in RBD binding to ACE2, viral infection, or virus neutralization by mAbs. The Alpha variant has a mutation at the 501 amino acid position in the RBD region (N501), which has been shown to increase binding affinity to human ACE2. There are three mutations within the RBD region in Beta variants. The Gamma variant is the same as that of the Beta variant, with the special case that K417 has been mutated to threonine instead of asparagine. In the Delta variant, two mutations have occurred in the RBD region that increased the affinity for ACE2. In the Omicron variant, 32 mutations occurred in the spike protein, 15 of which were located in the RBD region. In Lambda and Mu variants two and three mutations have occurred in the RBD region, respectively (Table 1) (6-8). SARS-CoV RBD consists of multiple conformation-dependent epitopes and can induce high-titer NAbs, showing that RBD is a crucial target for developing the SARS vaccine (9). RBD-based vaccines against SARS-CoV and MERS-CoV have been studied. Therefore, the design of RBD-based vaccines for SARS-CoV-2 can be suggested. Hence, RBD is a good target for SARS-CoV-2 vaccines (10). RBD is small, folds autonomously, and thus is a suitable candidate for protein subunit-based methods for SARS-CoV-2 vaccination (11). 

In this study, RBD was expressed by *E. coli*, and purified by a Ni Sepharose column. An animal study showed SARS-CoV-2 RBD-specific IgG-induced antibody responses. RBD-specific antibodies were also detected in the sera of mice and SARS-CoV-2-infected patients (for SARS-CoV-2 wild type and Delta variant).

## Materials and Methods

***Gene cloning***

*Escherichia coli* strains TOP10 (Invitrogen, USA) and BL-21 (DE3) (Novagen, USA) were respectively, employed for cloning and expression of the recombinant protein. The *E. coli* cells harboring recombinant plasmids were aerobically grown at 37 °C in Luria-Bertani broth (Merck, Darmstadt, Germany) with/without 50 µg/ml kanamycin (Sigma Co). Plasmid pET-28a (Novagen, USA) was employed as an expression vector.

***PCR and cloning***

Pishgaman Biology was used to synthesize the coding sequence (codon-optimized for *E. coli* strain BL-21(DE3)) for the RBD region, which spans residues 319–541 of the spike of the SARS-CoV-2 wild-type variant (Gen Bank accession number MN908947).

5′ATATATGAATTCGCCACCATGGTGAGGGT 3′ (forward) and 5′ ATTGATAAGCTTGAA GTTCACGCACTTGTTCT 3′ (reverse) were developed for PCR amplification of the SARS-CoV2 RBD. Primers introduced *Hin *dIII and *Eco RI* restriction sites on 3′ and 5′ ends, respectively. The initial denaturation temperatures were 95 °C for 5 sec, 30 cycles of 94 °C for 20 sec, 56 °C for 30 sec, and 72 °C for 30 sec accompanied by an ultimate extension of 72 °C for 5 min. Rapid digest *Hin *dIII and *Eco *RI enzymes (Fermentas Co) digested PCR products.

The purified PCR product of the RBD SARS-CoV-2 gene was ligated to the pET28 (a) vector. To transform in competent *E. coli* TOP10 cells, the ligation product was employed. Luria-Bertani not broth (LB) agar plate containing 50 μg/ml kanamycin was used to culture the cells. Colony PCR and restriction digestion were used to analyze the colonies. 

***Expression and purification of recombinant RBD SARS-Cov-2***

The recombinant plasmid was transformed to chemically competent *E. coli* BL21 (DE3) expression host cells through heat shock and spread onto an agar plate with kanamycin (50 μg/ml ) to select colonies that incorporated the plasmids successfully. 

Recombinant cells harboring pET28 (a)-RBD plasmid were screened on selective LB agar plates which were supplemented with kanamycin. A positive clone was chosen and grown for expression protein overnight in LB medium with 5 µg/ml kanamycin at 37 °C, shaking at 180 rpm. Overnight culture (500 µl) was added to 50 ml LB medium with 50 µg/ml kanamycin at 37 °C, shaking at 180 rpm. 1 mM isopropyl-β-D-galactopyranoside (IPTG; Fermentas Co.) was added to the culture medium and incubated for 16 hr at 37 °C after OD_600 _reached 0.6, expression was done in large-scale 50 ml culture and the bacterial precipitate was dissolved in 5 ml native buffer (50 mM NaH_2_ PO_4_ and 300 mM NaCl, pH 8) and was blended completely for analyzing the solubility of the protein. Also, sonication (6 x 10 sec with 10 sec pauses at 200–300 W) and lysozyme (1 mg/ml ) were used for complete cell lysis. Supernatant with soluble protein was collected after centrifugation at 13000 g for 10 min; the pellet was re-suspended in a denaturing buffer (100 mM NaH_2_ PO_4_, 10 mM Tris. HCl, and 8 m urea, pH 8) and incubated at 37 °C for 1 hr. The samples underwent analysis on 12% SDS-PAGE gel. The lysate was centrifuged (15 min, 10000 g, 4 °C) after cell disruption, and the supernatant was used on a Nickel–nitrilotriacetic acid (Ni-NTA) affinity chromatography column (Qiagen). The purification stages were done based on the guidelines of the manufacturer. Lysis buffer was used to equilibrate the column, and the protein solution was transferred onto the column at a 0.5 ml/min flow rate. By washing the column with washing buffer (100 mM NaH_2_ PO_4_, 10 mM Tris-Cl, 8 M urea pH 5.9), the impurity was removed two times. Elution buffer (100 mM NaH_2_ PO_4_, 10 mM Tris-Cl, 8M urea) was used to elute the protein at pH 4.5. The Bradford method with BSA (bovine serum albumin) determined the protein concentration.

***Western blotting to confirm the expressed protein***

Western blotting was used to assess recombinant proteins. The recombinant protein was separated by SDS-PAGE 12% and electro-transferred to the PVDF membrane (Roche). Non-fat skim milk (5%) in TBS buffer (50 mM Tris-Cl, 150 mM NaCl, pH = 7.5) with 0.05% Tween 20 (37 °C, 2 hr) was used to block the membrane. Anti-poly His-tag antibody (1:2000 Roche – Sigma Co) was used to incubate the membrane. The membrane was immersed in 3, 3’-Diaminobenzidine tablet (DAB Reagents; Sigma) for signal development.

***Mice immunization***

All study procedures were performed by international ethical standards, approved by the Ethics Committee of Islamic Azad University Science and Research Branch (2021-06-16/ IR.IAU.SRB.REC.1400.076).

A total of 10 BALB/c mice (female, 6–7 weeks old, Razi Institute, Tehran, Iran) were assigned to control and experimental groups (5 mice per each group), randomly, to indicate the recombinant RBD SARS-CoV-2 antigenicity. The animals were immunized four times. All mice in experimental groups were first vaccinated subcutaneously with 15 μg of refolded protein mixed with the same amount of Freund’s complete adjuvant (Sigma Co). The second and third doses were provided subcutaneously as boosters of 15 μg protein with incomplete Freund’s adjuvant (at 15-day intervals). The final dose was intra-peritoneally injected with 5 μg of the protein without adjuvant. Also, PBS with adjuvant was used for the control group (the same as the immunized animals). Serum samples were collected after the second and the fourth immunization to indicate antibody titers.

***Identification of serum antibodies against the RBD in mice and patients using an ELISA assay***

To coat polystyrene microplates, recombinant RBD (PBS as a control) was applied at a final concentration of 1 μg/ml in 100 µl coating buffer (64 mM Na_2_CO_3_, 136 mM NaHCO , NaN_3,_ pH 9.8) for 2 hr at room temperature. Then, the plates were rinsed three times with PBST having 0.1% Tween-20; the nonspecific sites were blocked with 5% skimmed milk solution (w/v) in PBST and stored at 37 °C for 1 hr. Patient sera (dilution 1:10 to 1:1000) and serially diluted mouse sera (dilution 1:100 to 1:24000) were added and incubated for 1 hr at 37 °C; the plates were then washed three times with PBST. Anti-human IgG HRP conjugate (Razi Biotech-Iran) and anti-mouse IgG HRP-conjugated antibodies (Sigma Co) were employed as secondary antibodies (diluted 1:2,000 in PBST and 1:10,000, respectively). Then, 100 μl of citrate buffer with 0.5 mg/ml of O-phenylene diamine dihydrochloride and 20 μl of hydrogen peroxide 30% were transferred to every well after washing and kept at 37 °C in the dark. The reaction was discontinued at 100 μl of sulfuric acid (2.5 M) when the solution color changed. On a microplate reader (Bio-Rad), the OD was measured at 492 nm.

Twenty COVID‐19 patients were admitted to the ICU of Baqiyatallah Hospital of Tehran (for SARS-CoV-2 wild type variant) and Nabiakram Hospital of Zahedan, Iran (for delta variant). They were recognized to be SARS‐CoV-2 positive by real-time polymerase chain reaction (RT-PCR). The control and patient groups were chosen randomly; both had 20 to 80 years of age. Written informed consent was obtained from all patient and healthy control participants. The study was confirmed by the Research Ethics Committee of Baqiyatallah and Nabiakram Hospitals.

To assess the potential immunogenicity of the S protein RBD as a human vaccine, serum samples of 20 patients (those infected with SARS-CoV-2 and recovered; SARS-CoV-2 wild type and delta variant) and 20 healthy donors were obtained. ELISA was used to detect the binding of the serum antibody to RBD, as previously described.

Serum was taken from the blood samples of every animal group (blood was transferred to vials and left to clot for 30 min, and then serum was obtained by centrifugation) and frozen at –70 °C. The serum samples were provided and then pooled to be immunologically analyzed.

***SARS-CoV-2 neutralizing antibody test kit***

The NAbs in immunized mice were measured by the SARS-CoV-2 neutralizing Ab ELISA kit (Pishtaz Teb, Iran, http://pishtazteb.com) according to the manufacturer’s protocol.

***Detection*** ***of inactive*** ***SARS-CoV-2***

The binding of sera mouse 1000 ng inactive SARS-CoV-2 was detected with ELISA as described above.

10^6^ PFU of SARS-COV-2 virus, which was amplified in Vero cells and inactivated, using temperature, was procured with a concentration of 100 µg from the Pasture Institute of Iran. After optimization *in vitro*, the concentration of 1000 ng of the inactivated virus was selected for the ELISA test. And DEME medium containing Vero cells was considered as a negative control. 1000 ng of inactive SARS-CoV-2 was coated in 100 μl of coating buffer on an ELISA plate and serum of mice vaccinated with recombinant RBD was added to each well in 1:100 dilutions with PBST. 

***Statistical analyses***

The ELISA outcomes for antibody responses were compared between immunized and non-immunized bunches and SARS-CoV-2 neutralizing antibodies utilizing SPSS 24 and one-way ANOVA. Contrasts were considered critical at *P*<0.05.

## Results

***PCR amplification and confirmation of pET-28a plasmid containing RBD gene***

**Figure 1:**
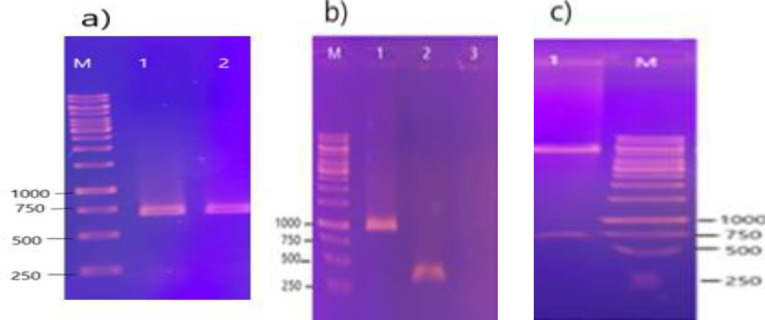
Analysis of PCR and cloning of RBD Gene. a) Amplification of SARS-CoV-2 RBD gene with the pfu enzyme on 1% agarose gel. M, DNA marker 1 kb; 1 and 2 the RBD gene (672 bp). b) PCR products from the RBD gene (=972 bp) by universal T7 primers. 1 positive sample; 2 negative samples (pET28a plasmid without insert); 3 negative control; M DNA marker 1 kb; c) Electrophoresis of the restriction enzyme digest (672 bp) by *Hin* dIII and *Eco *RI enzymes: 1, RBD fragment, M, 1kb

PCR amplification was observed on 1% agarose gel with a size of 672 bp (Figure 1a), and the PCR product was ligated into a pET28a vector. The plasmid extraction results are illustrated in (Figures 1b, 1c). PCR was done with T7 universal primers and digested by restriction enzymes *Eco RI* and *Hin *dIII, to ensure the RBD gene presence in the pET28a plasmid. The RBD fragments 672 bp and 972 bp were detected on 1% agarose gel. 

***Expression and purification of the recombinant protein***

**Figure 2:**
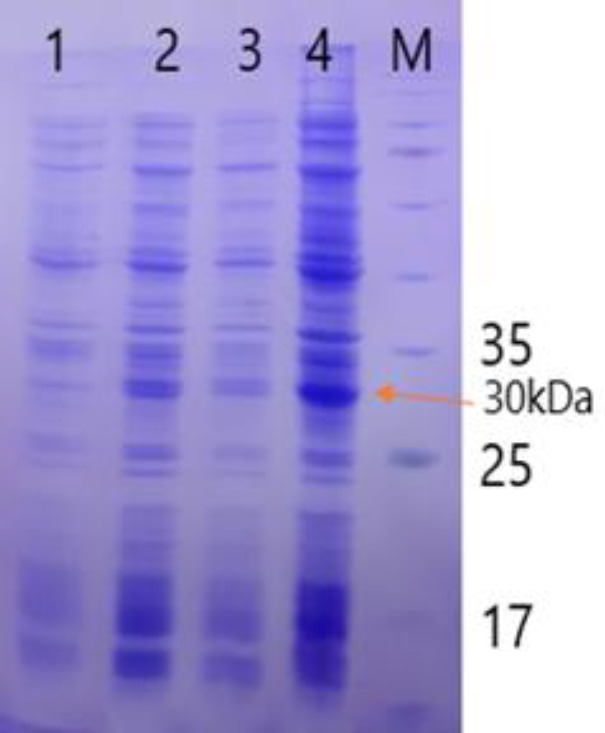
SDS-PAGE analysis of total bacterial proteins containing pET28a-RBD (30 kDa) in different time courses. 1, before induction; 2-4, 16 hr after induction with IPTG, respectively; M, protein molecular weight marker

RBD: receptor-binding domain; IPTG: isopropyl-β-D-galactopyranoside

**Figure 3:**
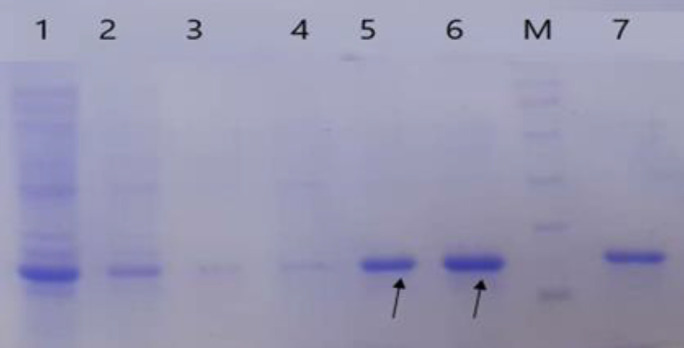
Purification of the recombinant RBD protein under denaturing condition by Ni-NTA column on SDS-PAGE 12%. 1 flow-through washing column with wash buffer (pH 5.9); 3 to 6 samples extracted from the column with elution buffer (pH 4.5); 7 extracted with MES buffer, M protein weight marker

We transformed the positive recombinant plasmid into *E. coli* BL21 (DE3), the host. The recombinant RBD protein expression with 6x His-tag (C-terminal) was analyzed on 12% SDS-PAGE (Figure 2). The recombinant protein generated as Inclusion Bodies (IB) was solubilized subsequently by 8 M urea. Under denaturing conditions, purification of the recombinant protein was carried out by Ni-NTA affinity chromatography (Figure 3). The purified recombinant protein was estimated through the Bradford method; it was found that the RBD concentration was 176 μg/ml.

***Western blotting to confirm the expressed protein***

**Figure 4:**
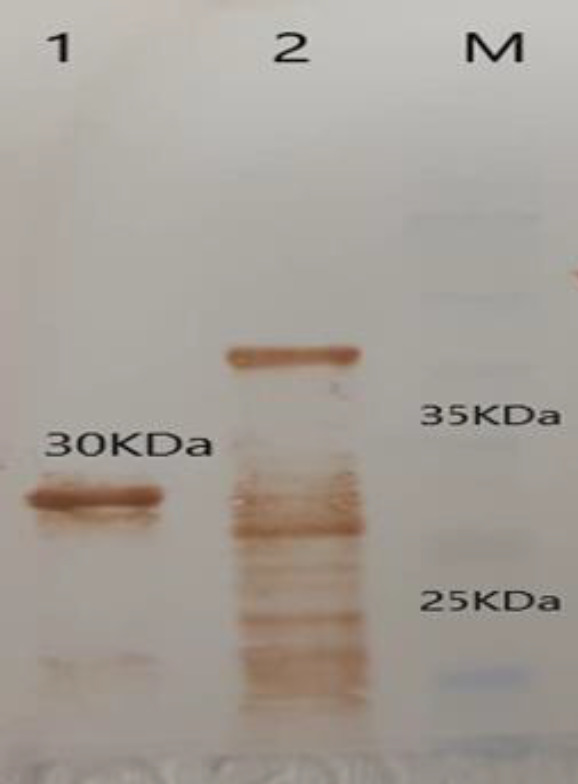
Analysis and confirmation of RBD purified recombinant proteins using Western blot. 1 RBD purified protein samples; 2 positive control; M protein weight marker

The accuracy of expression of recombinant RBD (30 kDa) protein was approved by an anti-poly His-tag antibody (Figure 4).

***Identification of serum antibodies against the RBD in mice using an ELISA assay***

**Figure 5:**
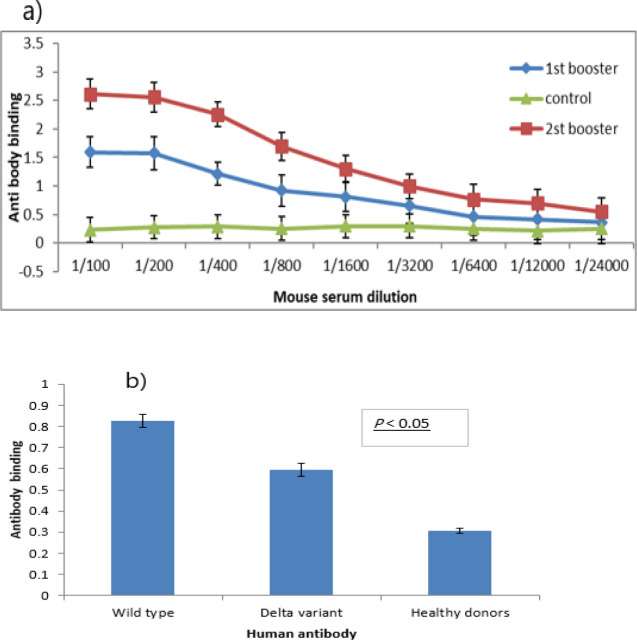
Serum antibody response against the spike protein RBD in mice and patients. a) Every mouse was immunized with 15 μg recombinant RBD proteins, compared with the control group. Serum antibody binding was estimated as absorbance at 450 nm. b) Serum samples were collected from 20 healthy donors and 20 patients infected with SARS-CoV-2 and recovered. Antibody binding to the RBD was recognized using 1:5 diluted sera by ELISA. Data are presented as mean ± SEM. P-values (P<0.05) were indicated by one-way ANOVA

The samples of blood were obtained, and IgG antibody titers were assessed. Indirect-ELISA approved that the changes in the antibody titer after every booster dose in the experimental group compared with the control group were significant. The impact of the number of booster immunization was analyzed through repeated measurements. The findings showed that the rise in antibody titer after every administration of the booster dose was significant (*P*<0.05). The level of this titer in the second blood sampling (dilutions 1:100 to 1:400) was much greater than that of the first blood in comparison with the control group (Figure 5a).

***SARS-CoV-2 antibodies in patient serum samples binding to RBD***

An immunoassay was used to approve that the RBD expressed in *E. coli* was detected by human SARS-CoV-2-specific antibodies. Recombinant RBD-bound microspheres were incubated with sera tested negative (n=20) or positive (n=20) in a clinical SARS-CoV-2 serology assay. RBD-specific IgG antibodies were recognized in all sera of patients with SARS-CoV-2 antibody-positive. The binding rate of human SARS-CoV-2-specific antibodies (wild Type) was higher than with the Delta strain (Figure 5b).

***SARS-CoV2 neutralizing antibody kit***

**Figure 6:**
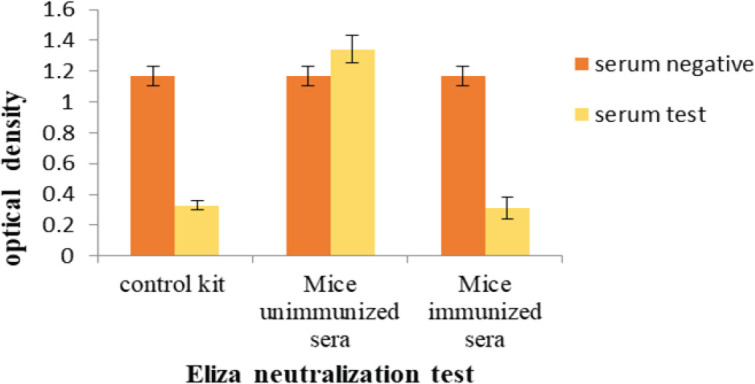
Inhibition of the binding of the RBD to ACE2. The sera of immunized mice could effectively block the binding of the RBD to the ACE2 (*P*<2.5)

RBD: receptor-binding domain; ACE2: angiotensin-converting enzyme 2

For the blocking activity of the RBD binding to the ACE2 receptor, we tested sera from immunized mice. RBD–ACE2 positivity was detected in immunized compared with unimmunized mice (Figure 6).

***Detection of inactive SARS-CoV-2***

**Figure 7:**
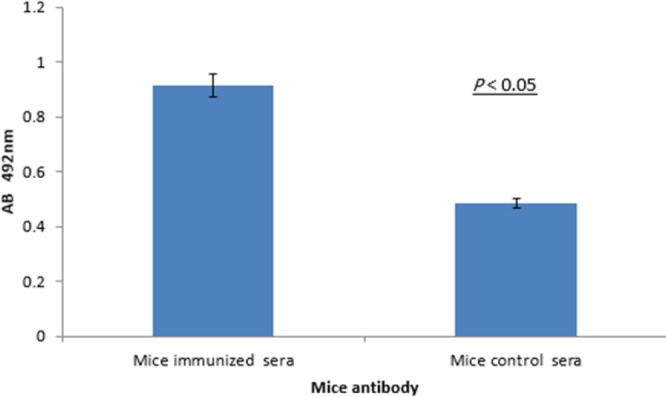
Identification of the inactive virus by immunized mouse serum compared with unimmunized mouse serum. Indirect-ELISA confirmed the significance (*P*<0.05) of the obtained result

The 1000 ng inactive SARS-CoV2 (wild type) was detected by RBD-immunized mouse serum (dilute 1:10 to 1:1000) antibodies (Figure 7).

**Table 1:** SARS-CoV-2 variants of concern (VoCs), variants of interest (VoIs), and Mutations in the receptor binding domain (RBD)

Variant category	Variants	RBD Mutations	First Detection
Variant of Concern (VOC)	Alpha Beta Gamma Delta Omicron	N501Y K417N, E484K, N501Y K417T, E484K, N501Y L452R, T478K G339D S371L S373P S375F K417N N440K G446S S477N T478K E484A Q493K G496S Q498R N501Y Y505H	The United Kingdom/ December 2020 South Africa / October 2020 Brazil / November 2020 India / October 2020 South Africa /November 2021
Variant Of Interest (VOI)	Lambda Mu	L452Q, F490S R346K E484K N501Y	Peru, South America/ December 2020 Colombia, South America

PCR: polymerase chain reaction; RBD: receptor-binding domain

RBD: receptor-binding domain

## Discussion

The SARS-CoV-2 pandemic has become a world catastrophe posing a major threat to the economy and the health system of human societies. Therefore, the production of an efficient vaccine is essential to control the SARS-CoV-2 pandemic. In this paper, RBD was expressed in *E. coli* and purified by Ni affinity chromatography. The product yield of RBD was 176 µg/ml by flask culture. The results of our experiments showed: 1) The serum of the immunized mouse was able to detect the protein RBD expressed in the bacterium *E. coli*. 2) The inactive virus SARS-CoV2 was detected by antibodies within the serum of immunized mice. 3) Serum antibodies from individuals recovered from Covid 19 (wild type and delta variant) reacted to the expressed protein. 4) When using the neutralizing antibody kit, antibodies produced by immunized mice prevented RBD from binding to ACE2. This kit is employed to diagnose people with Covid 19.

Various vaccines are proposed for controlling COVID-19, such as nucleic acid vaccines, subunits, an adenovirus-based vector vaccine, virus-like particle vaccines, and inactive and live-attenuated vaccines(12). Benefits of subunit vaccines include being secure to utilize in immunosuppressed patients, cannot cause illness state, due to decontaminated antigenic component, and fewer chances of a side-effect (13). According to published articles, both SARS-CoV-2 and SARS-CoV bind to the human ACE2 via RBD. Previous studies have shown the development of efficient and safe vaccine candidates against SARS-CoV based on RBD and screened Nabs, applying them as antigens (14). The RBD of SARS-CoV is expressed in *E. coli* and provides defensive immunity (15, 16). Different expression platforms are accessible, such as microbial systems like* E. coli*, different yeasts, mammalian cells, insect cells, and plants (17). Various eukaryotic expression systems, including mammalian cells(18-20), insect cells(18, 21), yeast (22, 23) and plants (24, 25) have been utilized to express the recombinant RBD protein. In several studies, RBD has been expressed in *E. coli*, such as the use of CyDisCo to get recombinant SARS-CoV-2 spike RBD that folded correctly with disulfide bonds in *E. coli* (26). The product yields of RBD-1 in *E. coli* and RBD-2 in mammalian cells (HEK-293T) were 13.3 and 5 mg/L, respectively. The structure of RBD-1 with RBD expressed through HEK293 cells (RBD-2 ) was compared. The results showed that the secondary and tertiary structures of RBD-1 were largely preserved. In addition, RBD-1 can potently bind to ACE2 (27). Fusion CRMA-RBD protein combined with FH-002C-Ac adjuvant can effectually induce neutralizing antibody titers (28). Recombinant RBD was expressed using a prokaryotic expression system. The results showed that non-glycol RBD could induce neutralizing antibody production (29). RBD-C9R expression in *E. coli* shows that antibodies produced by RBD-C9R in mice interacted with human ACE2 and recognized the commercial spike protein expressed from mammalian cells (30).* E. coli* is a broadly applied system for recombinant generation of proteins because of its fast growth and cost-efficiency, as well as access to extended molecular manipulation tools. Numerous vaccine antigens are produced in *E. coli*. Nevertheless, the expression systems of *E. coli* generally do not render post-translational modifications (PTMs), like glycosylation(17). The use of eukaryotic cells, due to the long production time, average efficiency, and high cost, makes them not suitable candidates for medical and clinical needs (30). The remarkable thing about the RBD domain (Arg319-Phe541) is the location of two N-glycoside bonds (Asn331 and Asn343), which are outside the motifs necessary for interaction with the human ACE2 receptor. The results of studies have shown that glycosylation is not essential for the induction of protective immunity using RBD (28). In this study SARS-CoV2 RBD despite having two N-glycosylation bonds was well expressed and purified in *E. coli*. Another finding was the identification of antibodies in the serum of people infected with delta variant, through recombinant RBD protein wild type variant. Therefore, this recombinant RBD protein could be commercially available for vaccine production on a global scale. In summary, our findings highlight that recombinant RBD domain induces protective immunity by inducing antibodies against RBD. Therefore, it can be used as a protective vaccine against SARS-CoV-2.

## Conclusion

The results obtained during this study confirm the immunogenicity of recombinant SARS-CoV-2 RBD expressed in *E. coli*. Therefore, RBD can be considered a suitable candidate for the production of recombinant SARS-CoV-2 vaccine which will trigger a humoral immunologic response. RBD may additionally be accustomed to diagnosing serum SARS-CoV-2 infection.

## Authors’ Contributions

ZR Conceived the study and design, processed and collected data, performed experiments, and wrote the manuscript. ShN developed the theory. RD and FS Reviewed the manuscript. JA Directed and managed the study, and proofread the manuscript. All authors read and approved the final manuscript.

## Fundind Statement

The authors declare no specific funding for this work.

## Etical Approval

 All applicable international, national, and/or institutional guidelines for the care and use of animals were followed. All study procedures were performed following international ethical standards and approved by the Ethics Committee of Islamic Azad University-Science and Research Branch (2021-06-16/ IR.IAU.SRB.REC.1400.076).

## Human and Animal Rights

All protocols of the study were approved by the institutional animal ethics committee of Baqiyatallah University of Medical Sciences which follows the NIH Guidelines for the Care and Use of Animals.

## Conflicts of Interest

The authors declare there are no competing interests.
